# Supplementary material for: Taking a critical stance towards mixed methods research: A cross-disciplinary qualitative secondary analysis of researchers’ views
Source: PLoS One. 2021 Jul 9;16(7):e0252014. doi: 10.1371/journal.pone.0252014 (PMC8270395; doi:10.1371/journal.pone.0252014)
Supplement: Attachment — Submitted filename: Review_PONE-D-20-2297_2020-09-23_submitted.pdf [file pone.0252014.s001.pdf]

Reviewer Comments
***PLOS ONE* Manuscript Number: PONE-D-20-22907**
DUE: 23 September 2020

Summary of Research:
The work presented in this manuscript addresses criticisms of mixed methods research based on authors' secondary analysis of semi-structured interviews with a cross-national sample of 42 researchers across the social scientific disciplines of education, nursing, psychology, and sociology; findings, conclusions, and implications for scholarship, and researchers' views within those scholarly fields that conduct and/or critique so-called 'mixed methods' research, are discussed, leading the authors to make four overarching recommendations, including that critical accounts be incorporated into "the pedagogy of mixed methods" (p.2).

Two research questions are posed, and answered: "(RQ1) What criticisms of the mixed methods field are made by researchers in education, nursing, psychology, and sociology? and (RQ2) What differences and similarities can be identified in the criticisms reported by researchers working in different disciplines?" (p.5,ln.67-70).

Claims & Conclusions:
Based on their secondary analysis of empirical data in the form of open-ended interviews – interviews, which were administered in a primary study on the closely related, yet (as authors claim) distinct, question of "researchers' views on the quality of mixed methods research" (p.5) – authors identify 11 discrete "themes" (p.8) of criticisms of mixed methods research; based on their choice to deductively group observed themes of criticisms into four existing categorical domains from Creswell [22], a prominent mixed methods researcher and methodological commentator, the authors' apparent conceptual claim is that criticisms of mixed methods according to researchers' views within these disciplines can be comprehensively, and correctly, classified into the particular categories of *essence, philosophy, procedure*, and *politics* (per Creswell).

Authors state that to their knowledge, theirs is "the first empirical study that has addressed the topic of criticisms of mixed methods as a field" (p.27). They thus contextualize the study as a contribution to 'the mixed methods literature,' recognized and defining itself as such, across multiple social scientific disciplines; since the study builds an explicit criticism-by-discipline analysis (RQ2), it also stands to make contributions to single-discipline literatures, especially in sociology since a key aspect of authors' findings concern the disciplinary distinctions that appeared to emerge from the sociologists in their sample.

A main result from this study according to authors is the identification of "two criticisms not previously mentioned in the literature": the "superficial presentation of pragmatism in the mixed methods literature" and "description of procedures that are not necessarily in line with actual research practice." (p.25)

Four final claims are made concerning "operationalization" (p.27), "conceptualization" (p.27), cross-disciplinary "awareness" (p.28), and what authors refer to as "pedagogy" in the Abstract but, notably, do not discuss as such, or at much length, in the manuscript itself (see p.28, ln.592-599, where "educational models" are mentioned, but not "pedagogy").

Global Comments:

This is an interesting study that provides a more transparent window into some of the recurring debates within and among mixed methods researchers and their critics and detractors, but perhaps more importantly, interview data reveals aspects of mixed methods critiques that have not been as explicitly or formally stated in the published research literature, and the findings raise some important operational, conceptual, professional, and practical implications. However, the credibility and validity of the work presented in this manuscript is hampered by the authors' editorializing recommendations and, at times, apparently unfounded interpretations of the empirical data. The mainly descriptive understanding gained from the study itself warrants dissemination without the need to overstep the bounds of what these empirics allow the authors to infer. The study authors describe was systematic, methodologically sound, and proceeded from a careful research design that affords reasonable confidence in the validity of descriptive results and makes the limits of generalizability and potential sources of bias clear. Yet, subjective interpretations beyond the data amount to a form of advocacy that the study at hand simply does not warrant.

Recommendation:
Based on my review of the work in its present form, I recommend that *PLOS ONE* request that authors **Revise & Resubmit, with Major Revisions**, Manuscript Number: PONE-D-20-22907, in order to address this main weakness, with a focus on rethinking and rearticulating the nature of the implications of the findings in the current Discussion section (pp.23-29) and Abstract.

With the revisions I suggest below -- which I have confidence the authors will be able to address based on their thought, care, and clear writing already displayed in this first submission – the study stands to make an important empirical contribution across social scientific fields, and I would be available to review a revised version of the manuscript as needed.

Please note that I have included a list of References I encourage authors to consult, and which I reference in my comments below, at the end of my review.

Major Revision Issue
In my view, the major issue fundamental for the current study and essential points the authors would need to address before the manuscript can proceed boils down to: over-interpretation of the findings.

The authors aim to demonstrate what criticisms of the mixed methods field are made by researchers in education, nursing, psychology, and sociology (RQ1), and what differences and similarities can be identified in the criticisms reported by researchers working in different disciplines (RQ2). The data allows authors to present valid answers to these two research questions, which they do; however the data does not support authors' discussion of implications, nor the ultimate conclusions they draw about what can, or what ought, be inferred from those answers based on their empirical results.

→ Specifically, in the "Practical Implications" section (starts p.27), authors should consider how to attend, and then attend, to foundational assumptions about what defines 'science,' even among contested definitions within the social sciences, which they currently elide in the manuscript's present discussion. The present discussion inappropriately puts forth what amount to normative, prescriptive interpretations about the data rather than theorize results to explain how what they found either confirms or upends in some way the areas of social scientific consensus that do exist, and/or how findings contribute to a better or deeper understanding of unsettled or unstable assumptions about what 'counts' as science in the social sciences.

The entirety of the Practical Implications section seems to presume no readers, and perhaps no one conceivably, would take issue with the authors' assertion – which is implicit yet glaring in the manuscript as currently written – that mixed methods research may be somehow exempted from the basic definitional requirement of having some namable, even if not agreed up as 'objective,' criterion for evaluating the validity of mixed methods research put forth and understood as 'science' in even the most "pluralistic" sense. Specifically, in discussing the second asserted implication regarding "conceptualization" (p.27), authors state that "Having an open mind" and an "inclusive attitude towards these criticisms" will "allow us to benefit from the complexity, free expression of opinion, and intellectual vigor that characterizes this field" as a stance "to allow the methodology to evolve conceptually in day-to-day practice" – this reader is left wondering how prescribing the absence of a scientific criterion for mixed methods research would do much to advance debates in the field, let alone the credibility or a "pedagogy" to speak of for any social scientific concept of mixed methods research when the very condition of possibility for articulating any kind of "methodology" is some criterion for evaluating internal and external validity and some amount of systematicity. If the implication of this study is a prescribed criterion that amounts to little more than "free expression of opinion," then I fear the authors arrive, unnecessarily and far too soon, at a definitional limit between what can be claimed as properly 'scientific,' within even the most capacious concept of 'science,' and what must be differentiated as something else, and perhaps, though not necessarily, dismissed as mere opinion, rhetoric, or myth and, as disinformation, disavowed -- especially in a threatening 'post truth,' 'fake news,' 'fake facts' age. I encourage authors to see Hancock, Sykes & Verma (2018) in *Sociological Perspectives* for what may be a useful framework for organizing a more theoretical (prior to, and I suggest, rather than practical) response to the dilemmas raised by criticisms identified in this study.

By the end of this discussion (p28, ln.586), the authors leave one wondering about the fundamental project of articulating mixed methods "methodology" when their foregoing analysis seems to seek an escape route out of the rigors and systematicity demanded by even diverse concepts of 'method' and 'methodology,' regardless of whether and where a 'methodological' orientation sits on the positivistic quantitative-to-interpretive qualitative dichotomy, or a looser pluralistic spectrum if conceived as such.

→ Ultimately, what seems at issue, and most at stake, based on the criticisms found in this study, is incommensurability and divergent epistemologies, including the ethical stances and commitments that flow from divergent definitions and methods for articulating 'truth' and what counts as 'knowledge' and ultimately 'science,' rather than the more surface-level problems of legibility and translation. To better theorize the incommensurability dimension of their empirical findings, and to better specify the implications of their findings theorized as such, I suggest authors review Small's (2011) *Annual Review of Sociology* piece, which takes on the demands of commensurability and problems of incommensurability in mixed methods research directly.
My concerns in the same vein apply to authors' discussion stating that "knowledge is grounded in fostering a culture of criticism" (p.28, ln.599) – true, but according to some legibly named (and one would hope, valid) criterion. You cannot have criticism without criteria as a basis for mounting and then evaluating the critique. Otherwise, authors may advocate as necessity mistaking free speech ("the free expression of opinion" as they put it on p.27) for "criticism" of the rigorous and scientifically-generative kind.

I recommend revisiting Thomas Kuhn's *The Structure of Scientific Revolutions* as a useful theoretical touchstone for authors to address many of these challenges at once; Kuhn could help ground what I

would like to see as a "*Theoretical Implications"* section (rather than the presently prescriptive "Practical" implications section), which would engage a deeper discussion of how an unsettled criterion – including an unsettled consensus on whether such a criterion/criteria is possible, and/or necessary to strive for within social scientific mixed methods fields—has classically been a crucial way to define a community of scientific inquiry as both a social and scientific matter. Moreover, Kuhn presents a clear argument about what more precisely is meant by "paradigm" which would help significantly to theorize authors' discussion of the criticism concerning mixed methods as a "third paradigm" (starting p.13).

→ My final major concern with the Discussion and conclusions reached in the current iteration of this manuscript relate to authors' deployment of the term "pedagogy," which seems little more than a keyword used in the Abstract but then never meaningfully articulated in substance or theory in the manuscript itself. While there of course is some nuance and contestation about meaning, a reasonable consensus is that "pedagogy" refers to study of the method of teaching and teaching methods, and that the term means to encompass theoretical frameworks for how learning works. (see i.e., Murphy 2008; Galumba 2016). I wonder if "curriculum" may be a better term for what authors currently describe in the conclusion of the manuscript (p.28), which offers far too little substantive detail on the nature of this final recommendation but mentions "courses and workshops on mixed methods research." On this, please refer to Hancock, Sykes & Verma (2018) in *Sociological Perspectives,* who conclude by presenting empirical findings on the absence of mixed methods curricula and training in US doctoral sociology programs and then make specific, empirically-grounded recommendations about the need for mixed methods curriculum in the social sciences for "training the 21st century ethnographer" (p.325).

If authors aim to pursue "pedagogy" as a more ambitious and deeper theoretical recognition of the implications of the criticisms in researchers' views they identified in this study, they need to interrogate the distinct definitional realm a "pedagogy" (beyond expanding 'curriculum') would offer for more properly engaging the criticisms of mixed methods research, and especially those criticism that appear intractable given apparent limitations in current social scientific pedagogies. To this end, a brief foray into the literature on "pedagogy" is in order – see especially articulations of differences between so-called "scientific" / "humanist" / "liberal" / "vocational" / "critical" pedagogies (i.e., Galumba 2016); it may be that something akin to a pivot from "scientific" to "critical" pedagogy is actually what the authors are trying to argue for by the conclusion of their work here (I'm not sure, but this needs to be clarified).

Other Issues

Additional recommendations from the literature to bolster authors' theorization of thematic criticisms based on their empirical findings, specifically with respect to:

- *Criticism 6: Superficiality of pragmatism* (p.16): see Tavory & Timmermans (2013); and
- *Criticism 10: Mixed methods research is better than monomethod research* (p.20): see Sykes, Verma & Hancock (2018, p. 231-233) in *Ethnography* (specifically their "Theoretical Demonstration of the Problem" section starting p. 231, and Figure 1, p.232 representing what they articulate as "Breadth-depth optimization in mixed-methods research designs" as another way of expressing what authors identify as *Criticism 10* in the present study); note that Sykes, Verma & Hancock 2018 also present "An empirical demonstration of the problem" starting p.233, which authors may want to be aware of in stating that theirs is "the first empirical study that has addressed the topic of criticisms of mixed methods as a field" (p.27).

Summary & Conclusion
Overall, the authors have presented a methodologically sound study and described valid results, which will be of interest to a variety of social scientific disciplinary and inter-disciplinary fields wherein researchers continue to grapple with important dilemmas of mixed methods research, including articulation of a criterion, or criteria, for assessing and addressing criticisms of the various iterations of mixed methods "methodology"; however, as presented in the current the manuscript, authors' over-interpretation leading to their discussion of "Practical Implications" of empirical findings should undergo major revision to address what I see as a theoretical under-interpretation that is currently detrimental to the external validity of this research.

Thank you for the opportunity to review this manuscript.

Suggested References:

Galamba, Arthur. "Conflicting interpretations of scientific pedagogy." *Science & Education* 25, no. 3-4 (2016): 363-381.

Hancock, Black Hawk, Bryan L. Sykes, and Anjuli Verma. "The problem of "cameo appearances" in mixed-methods research: Implications for twenty-first-century ethnography." *Sociological Perspectives* 61, no. 2 (2018): 314-334.

Kuhn, Thomas S. *The Structure of Scientific Revolutions*. University of Chicago Press, (1962/2012).

Murphy, Patricia. "Defining pedagogy." *Pedagogy and practice: Culture and identities* (2008): 28-39.

Small, Mario Luis. "How to conduct a mixed methods study: Recent trends in a rapidly growing literature." *Annual review of sociology* 37 (2011).

Sykes, Bryan L., Anjuli Verma, and Black Hawk Hancock. "Aligning sampling and case selection in quantitative-qualitative research designs: Establishing generalizability limits in mixed-method studies." *Ethnography* 19, no. 2 (2018): 227-253.

Tavory, Iddo, and Stefan Timmermans. "A pragmatist approach to causality in ethnography." *American Journal of Sociology* 119, no. 3 (2013): 682-714.
