# Supplementary material for: Taking a critical stance towards mixed methods research: A cross-disciplinary qualitative secondary analysis of researchers’ views
Source: PLoS One. 2021 Jul 9;16(7):e0252014. doi: 10.1371/journal.pone.0252014 (PMC8270395; doi:10.1371/journal.pone.0252014)
Supplement: Attachment — Submitted filename: Response to Reviewers.docx [file pone.0252014.s002.docx]

Adam T. Perzynski, PhD

Academic Editor, PLOS ONE

Dear Dr. Perzynski,

We would like to thank you and the reviewers for your feedback on our manuscript entitled, “Taking a critical stance towards mixed methods research: A cross-disciplinary qualitative secondary analysis of researchers’ views,” and for the opportunity to submit a revised version of the manuscript. As requested, we have revised the manuscript based on your suggestions, and we believe that these revisions have improved the overall quality of the manuscript. To facilitate review, we have created a table detailing our revisions, with our line-numbered-responses to each reviewer and every comment. We have also highlighted in yellow the changes made in the manuscript.

| **EDITOR** | | |
| --- | --- | --- |
|  | **Comment** | **Response** |
| 1 | In addition to the reviewer comments, I have a major concern about the multiple correspondence analysis. How reproducible are the MCA results? I would suggest that the authors more explicitly acknowledge that this part of the paper is more exploratory than thematic/content analysis. | Thanks for raising this issue. In the limitations section, we have added a statement explaining that MCA is an exploratory method not appropriate for testing hypotheses and that the findings regarding RQ2 should be subject to further investigation (lines 765-771). Besides acknowledging the limitations of MCA, we have also noted that the method helped us to generate a visual representation of the underlying structure of the data, and to improve the interpretation of the qualitative data (lines 781-785). |
| 2 | I strongly agree with Reviewer 1's concern about use of the term pedagogy and the associated text in the discussion. The dissimilarity between practice and textbook descriptions of mixed methods is notable, but the authors should be more precise with use of language here. | We have addressed this point in our responses to comment 6 (742-751). We have also clarified the explanation of criticism 9 (lines 487-489). |
| **REVIEWER 1** | | |
| 3 | However, the credibility and validity of the work presented in this manuscript is hampered by the authors’ editorializing recommendations and, at times, apparently unfounded interpretations of the empirical data (…) subjective interpretations beyond the data amount to a form of advocacy that the study at hand simply does not warrant (…) the data does not support authors’ discussion of implications, nor the ultimate conclusions they draw about what can, or what ought, be inferred from those answers based on their empirical results. | We have addressed these points in the following responses. |
| 4 | Specifically, in the “Practical Implications” section (starts p.27), authors should consider how to attend, and then attend, to foundational assumptions about what defines ‘science,’ even among contested definitions within the social sciences, which they currently elide in the manuscript’s present discussion. The present discussion inappropriately puts forth what amount to normative, prescriptive interpretations about the data rather than theorize results to explain how what they found either confirms or upends in some way the areas of social scientific consensus that do exist, and/or how findings contribute to a better or deeper understanding of unsettled or unstable assumptions about what ‘counts’ as science in the social sciences (…) I recommend revisiting Thomas Kuhn’s The Structure of Scientific Revolutions as a useful theoretical touchstone for authors to address many of these challenges at once; Kuhn could help ground what I would like to see as a “Theoretical Implications” section (rather than the presently prescriptive “Practical” implications section), which would engage a deeper discussion of how an unsettled criterion – including an unsettled consensus on whether such a criterion/criteria is possible, and/or necessary to strive for within social scientific mixed methods fields—has classically been a crucial way to define a community of scientific inquiry as both a social and scientific matter. Moreover, Kuhn presents a clear argument about what more precisely is meant by “paradigm” which would help significantly to theorize authors’ discussion of the criticism concerning mixed methods as a “third paradigm” (starting p.13) (…) Ultimately, what seems at issue, and most at stake, based on the criticisms found in this study, is incommensurability and divergent epistemologies, including the ethical stances and commitments that flow from divergent definitions and methods for articulating ‘truth’ and what counts as ‘knowledge’ and ultimately ‘science,’ rather than the more surface-level problems of legibility and translation. To better theorize the incommensurability dimension of their empirical findings, and to better specify the implications of their findings theorized as such, I suggest authors review Small’s (2011) Annual Review of Sociology piece, which takes on the demands of commensurability and problems of incommensurability in mixed methods research directly. | We are grateful for your suggestions and references. We have rewritten the section about the practical implications of the study (now entitled “Theoretical implications of the study”), including your suggestions. In this section, we have extensively described the theoretical implications of our findings (lines 685-751). We have highlighted how, due to substantial differences in philosophical and theoretical viewpoints and their membership to different research cultures, researchers in this field sometimes disagree on foundational issues. We have also argued that this divergence is consistent with Denscombe's use of the theoretical notion of "communities of practice" as a basis for describing the mixed methods field. Inspired by Thomas Kuhn's definition of paradigms as shared beliefs in a research community, Denscombe suggested this notion in 2008 to characterize the mixed methods field as a conglomerate of multiple research communities shaped by the disciplinary principles and values prevailing in each community. We have discussed how our findings are consistent with Denscombe's approach. Concerning the incommensurability problem, we have cited Small (2011) to clarify that the diversity of views in the mixed methods field we identified in our study makes complete agreement and communication among researchers impossible. To address this problem, we have argued that, in line with Kuhn's views, conflicting perspectives can be reconciled to a certain extent (lines 709-712). Finally, we have described how being aware of the divergence of viewpoints in the field could help researchers improve the quality of mixed methods research (lines 729-741). |
| 5 | The entirety of the Practical Implications section seems to presume no readers, and perhaps no one conceivably, would take issue with the authors’ assertion – which is implicit yet glaring in the manuscript as currently written – that mixed methods research may be somehow exempted from the basic definitional requirement of having some namable, even if not agreed up as ‘objective,’ criterion for evaluating the validity of mixed methods research put forth and understood as ‘science’ in even the most “pluralistic” sense. Specifically, in discussing the second asserted implication regarding “conceptualization” (p.27), authors state that “Having an open mind” and an “inclusive attitude towards these criticisms” will “allow us to benefit from the complexity, free expression of opinion, and intellectual vigor that characterizes this field” as a stance “to allow the methodology to evolve conceptually in day-to-day practice” – this reader is left wondering how prescribing the absence of a scientific criterion for mixed methods research would do much to advance debates in the field, let alone the credibility or a “pedagogy” to speak of for any social scientific concept of mixed methods research when the very condition of possibility for articulating any kind of “methodology” is some criterion for evaluating internal and external validity and some amount of systematicity. If the implication of this study is a prescribed criterion that amounts to little more than “free expression of opinion,” then I fear the authors arrive, unnecessarily and far too soon, at a definitional limit between what can be claimed as properly ‘scientific,’ within even the most capacious concept of ‘science,’ and what must be differentiated as something else, and perhaps, though not necessarily, dismissed as mere opinion, rhetoric, or myth and, as disinformation, disavowed -- especially in a threatening ‘post truth,’ ‘fake news,’ ‘fake facts’ age. I encourage authors to see Hancock, Sykes & Verma (2018) in Sociological Perspectives for what may be a useful framework for organizing a more theoretical (prior to, and I suggest, rather than practical) response to the dilemmas raised by criticisms identified in this study (…) By the end of this discussion (p28, ln.586), the authors leave one wondering about the fundamental project of articulating mixed methods “methodology” when their foregoing analysis seems to seek an escape route out of the rigors and systematicity demanded by even diverse concepts of ‘method’ and ‘methodology,’ regardless of whether and where a ‘methodological’ orientation sits on the positivistic quantitative-to-interpretive qualitative dichotomy, or a looser pluralistic spectrum if conceived as such (…) My concerns in the same vein apply to authors’ discussion stating that “knowledge is grounded in fostering a culture of criticism” (p.28, ln.599) – true, but according to some legibly named (and one would hope, valid) criterion. You cannot have criticism without criteria as a basis for mounting and then evaluating the critique. Otherwise, authors may advocate as necessity mistaking free speech (“the free expression of opinion” as they put it on p.27) for “criticism” of the rigorous and scientifically-generative kind. | As explained above, we have rewritten the “Practical Implications” section, which is now entitled “Theoretical implications”. We do not think that the existence of divergent views in the mixed methods field is incompatible with the purpose of defining core criteria for evaluating the quality of mixed methods research. We also don’t think that this idea is implicit in our manuscript. On the contrary, according to Fàbregues and Molina-Azorin (2007), despite the inevitable diversity of views in the field, it is still possible to reach a consensus on a parsimonious set of core criteria that are broad and flexible enough to accommodate different researcher and study contexts. This argument has also been supported by prominent authors in the field such as Bryman (2014) and Creswell and Plano Clark (2018). We have not included this argument in our manuscript . However, concerning quality, in lines 730-741, we have argued that being aware of the diversity of views in the field could help researchers improve the execution of mixed methods studies by facilitating the integration process and overcoming methodological tokenism. We thank the reviewer for suggesting the article by Hancock, Sykes & Verma (2018), which we have included in this section. |
| 6 | My final major concern with the Discussion and conclusions reached in the current iteration of this manuscript relate to authors’ deployment of the term “pedagogy,” which seems little more than a keyword used in the Abstract but then never meaningfully articulated in substance or theory in the manuscript itself. While there of course is some nuance and contestation about meaning, a reasonable consensus is that “pedagogy” refers to study of the method of teaching and teaching methods, and that the term means to encompass theoretical frameworks for how learning works. (see i.e., Murphy 2008; Galumba 2016). I wonder if “curriculum” may be a better term for what authors currently describe in the conclusion of the manuscript (p.28), which offers far too little substantive detail on the nature of this final recommendation but mentions “courses and workshops on mixed methods research.” On this, please refer to Hancock, Sykes & Verma (2018) in Sociological Perspectives, who conclude by presenting empirical findings on the absence of mixed methods curricula and training in US doctoral sociology programs and then make specific, empirically-grounded recommendations about the need for mixed methods curriculum in the social sciences for “training the 21st century ethnographer” (p.325). | Thanks for pointing this out. Following the reviewer’s recommendation, we have replaced the word *pedagogy* with *teaching of* and *curriculum* in the abstract and in the text. We have also highlighted the fact that published syllabi do not address the topic of criticisms: “This last point is particularly important since the topic of criticisms is not included in any of the mixed methods syllabus exemplars published in the literature, including those by Earley (59), Christ [60], and Ivankova and Plano Clark [12]” (lines 748-751). |
| 7 | If authors aim to pursue “pedagogy” as a more ambitious and deeper theoretical recognition of the implications of the criticisms in researchers’ views they identified in this study, they need to interrogate the distinct definitional realm a “pedagogy” (beyond expanding ‘curriculum’) would offer for more properly engaging the criticisms of mixed methods research, and especially those criticism that appear intractable given apparent limitations in current social scientific pedagogies. To this end, a brief foray into the literature on “pedagogy” is in order – see especially articulations of differences between so-called “scientific” / “humanist” / “liberal” / “vocational” / “critical” pedagogies (i.e., Galumba 2016); it may be that something akin to a pivot from “scientific” to “critical” pedagogy is actually what the authors are trying to argue for by the conclusion of their work here (I’m not sure, but this needs to be clarified). | As noted above in our response to comment 6, we have replaced this word with the terms *teaching* and *curriculum* in the new version of our manuscript. |
| 8 | Criticism 6: Superficiality of pragmatism (p.16): see Tavory & Timmermans (2013) (…) Criticism 10: Mixed methods research is better than monomethod research (p.20): see Sykes, Verma & Hancock (2018, p. 231-233) in Ethnography (specifically their “Theoretical Demonstration of the Problem” section starting p. 231, and Figure 1, p.232 representing what they articulate as “Breadth-depth optimization in mixed-methods research designs” as another way of expressing what authors identify as Criticism 10 in the present study); note that Sykes, Verma & Hancock 2018 also present “An empirical demonstration of the problem” starting p.233, which authors may want to be aware of in stating that theirs is “the first empirical study that has addressed the topic of criticisms of mixed methods as a field” (p.27). | Thanks for providing these references. Since we have changed the wording of criticism 10 in response to a comment of reviewer 3, this criticism refers now to a tendency in some of the MMR literature to consider MMR as inherently superior to monomethod research, which is different to the type of criticism described by Sykes, Verna and Hancock (2018). Furthermore, we have made it clearer that our article addresses criticisms of how mixed methods have been developed and conceptualized by the mixed methods community rather than how mixed methods are implemented in practice (which is the type of criticism described by Sykes et al. (2018) when they allude to the bias that exists in mixed-methods studies when “sampling is incongruent within research designs”, p. 227). |
|  |  | We are grateful for your comments and suggestions that have helped to improve our manuscript. |
| **REVIEWER 2** | | |
| 9 | My concerns mainly revolve around making the language a little more accessible to the wide readership of PLOS by explaining terminology used, and providing additional detail so that readers may better understand the methods and findings. If it is possible (in terms of word limits), the manuscript would be enhanced if the authors utilized more quotations to illustrate their findings and allay any reader concerns that many of the quotations are coming from the same individuals. | We have addressed these points in the following responses. |
| 10 | Line 35: It would be helpful to readers unfamiliar with qualitative work for the authors to describe what is meant by “typologies.” | We have clarified the definition of typologies by adding the following sentence: “Typologies are used in the mixed methods literature as classifications of methodological features, such as the timing and priority of the quantitative and qualitative components and the stage at which integration is carried out” (lines 443-446). |
| 11 | Lines 74-75: Related to points #7 and #21 below, if the secondary data analysis was already “covered” by the IRB, why were participants recontacted? It appears from later in the manuscript that this contact was an opportunity for authors to collect additional information from participants? Please clarify. | In the member checking process of the original study, we recontacted participants to confirm that the data accurately represented their views. This step also gave them an opportunity to elaborate on what they stated in the interviews. We have made this clear in the description of the data collection procedures (lines 169-173). |
| 12 | Line 86: It would be helpful to readers if the authors explained what they meant by criterion sampling. Does this mean there were inclusion criteria? | As we explained in lines 147-150, criterion sampling was used to select participants that met two inclusion criteria. We have clarified this by adding the phrase “In the criterion sampling, participants fulfilled two inclusion criteria” (lines 147-148) and by citing Patton (2014) on the use of criterion sampling. |
| 13 | Line 105: This is the data collection procedure for the original study? Please clarify. | This section describes the data collection procedures used in the original study. This was described in lines 130-134: “In the following subsections, we provide a brief description of the sampling and data collection methods used in the original study, and of the procedures used in the secondary analysis of data”. To clarify the difference between the original study procedures and the later secondary analysis of the data, we have divided the Methods section in two sub-sections: “The original study” and “The secondary data analysis”. |
| 14 | Lines 106-107: There seems to be some missing text, and there is no (a). | Thanks for pointing this out. We added the fragment of text that was missing: “Questions from the interview guide were focused on the following: a) participant’s research background, (b) participant’s conceptualization of…” |
| 15 | Line 109 (two interviews): In other words, each participant was interviewed twice? Please clarify. | Each participant was interviewed once. We have clarified this by adding the following text: “and, in two cases, e-mail correspondence” (lines 167-168). |
| 16 | Related to Point #21 below, the fact that the authors were recontacted and given opportunity to clarify their responses needs to be described here in the data collection. Presumably, it was this opportunity to clarify that necessitated recontacting participants (point 2 above) and that two individuals turned down? Please clarify. | We have added in the data collection section the information on the member checking procedures followed (see our response to reviewer’s comment 11). All the participants agreed to participate in the member checking process. The two participants mentioned in lines 195-197 did not give permission to use their interview data for secondary analysis in subsequent research (this decision was mentioned in the reporting of the informed consent in the original study). For this reason, these two participants were excluded in this secondary analysis. We expect that the changes made in response to the reviewer’s comment 13 will clarify the distinction between the procedures of the original study and those used in the secondary analysis. |
| 17 | Lines 164-165. It would be helpful to readers unfamiliar with Creswell’s publication for the authors to provide more descriptive information about each of the four domains. For example, what do “philosophy” and “politics” refer to here? | We have added four parentheses containing a brief explanation of the meaning of each domain (lines 249-252). |
| 18 | Criticism #2 in Table 2: I think it should be “proponents.” | Thanks for pointing this out. We have corrected the word. |
| 19 | General concern about the quotations: The quotations are very helpful to illustrate points. Is there a way the authors could identify the individuals, may like “Educationalist #1” or “Sociologist #3” to allay readers’ concerns that all the quotations from sociologists, for example, are not coming from the same sociologist. | We have added identifiers to each of the quotes. The total number of participants quoted in the article is 23 out of 27. |
| 20 | Lines 257-259: the phrase “…presupposes a strong link between epistemology and method, whereas….” is unclear. Please clarify. | We made this statement clearer by adding the following text: “that is, the identification of the use of mixed methods with a particular epistemological or ontological view” (lines 347-348). |
| 21 | Lines 279-281: To help readers unfamiliar with the dominant rules of the 1980s, can the authors explain more about what the rules were. | We have clarified this by stating that the methodological literature of the 1980s tended to associate qualitative research with the constructivist paradigm and quantitative research with the positivist paradigm (lines 368-369). We note that that the word “rules” is taken from one participant’s quote. |
| 22 | Lines 312-315: For readers unfamiliar with these terms, the authors should explain what is meant by “pragmatism,” “eclecticism,” and “practicalism.” | The meaning of these terms is explained in the following sentence, in lines 403-406. We have replaced the adverb “thus” with “in this way” to make this clearer. |
| 23 | Line 343: The authors use the phrase “in some disciplines,” but from the quotation that follows it is not clear whether the participant was referring to entire disciplines or individual researchers. Please clarify. | Thanks for noting this. We have made it clear that the participant was referring to researchers in general. |
| 24 | Lines 393-406: It is a little concerning that the authors refer to participants in the plural but provide only one quotation. Is it possible to provide additional quotations to show more clearly to readers that multiple participants shared these views. | Thanks for pointing this out. We have added two additional quotes (lines 468-470 and lines 475-480). |
| 25 | Lines 427-429: It is interesting that some participants complained about the tendency for homogenized terminologies and procedures, while other participants complained about the variation in terms and lack of agreement (lines 202-206). I might have missed it, but this might be important to include in the discussion: that the criticisms can be contradictory, which might make them difficult to address in a manner that will please everybody. | Thanks for raising this point. In the Implications section, we have pointed out these contradictions to exemplify the disagreements among mixed methods researchers on foundational issues, owing to differences in philosophical stances and their membership to different research cultures. |
| 26 | Lines 448-466: The authors need to help readers understand what Figure 1 is showing and what correspondence analysis mapping is doing. What does 92.4% actually refer to? (line 453). What do the values of -1.5 to 1.5 mean on the axes. It will be really helpful to readers if the authors can explain more clearly why a specific discipline is placed where it is relative to the axes’ scales. | We have added to the findings section a detailed explanation on how to interpret Figure 1, and of the significance of eigenvalues and coordinates (lines 555-562) |
| 27 | Lines 486-487 “known for their criticisms”: Were the individuals known for their criticisms all from the same discipline? | These individuals were from the disciplines of sociology and education. We have clarified this in line 600. |
| 28 | Line 541 Limitations: The small numbers and the relatively small number of disciplines should be noted as limitations. | We added the following sentence in the limitations section: “Second, the transferability of our findings is limited by our decision to include only four disciplines while excluding the views of researchers working in other disciplines that also have a high prevalence of mixed methods studies, such as medicine, business, and information science. From our findings alone, it is not possible to infer how frequent these criticisms are and what types of criticisms may be more prevalent in each of the disciplines” (lines 760-765) |
| 29 | Line 541: It seems like a limitation that should be noted is that the sample was limited to researchers who have written on the methods of mixed-methods research, especially because one of the criticisms was that the people who are writing about mixed methods aren’t necessarily the ones who are really doing mixed methods research. | The rationale for including participants with methodological publications on mixed methods research is that they would be certain to have the knowledge of the nature and practice of MMR needed to provide a sufficiently informed opinion on MMR. Since all the participants, except two, had experience in carrying out mixed methods research, the inclusion of this type of participants should not be considered a limitation. We have added in Table 1 the attribute on the experience of conducting mixed methods research. |
| 30 | Line 547-548: The ability to provide additional insights needs to be included in the methods section. Is this what the two persons refused to do? | We have included this information in the data collection section (lines 169-173). In the methods section, we also made it clear that two participants refused their data to be used for secondary analysis (lines 195-199). |
| 31 | Line 567 “typologies”: Was this typologies of methods? Please clarify. | We have made it clear that this sentence refers to both mixed methods designs and procedures. |
| 32 | Maybe I missed it, but do the authors have ideas for future research in this area? | In the “Limitations, strengths, and possibilities for future research” section, we have added a paragraph with a few ideas for future research (lines 791-804). |
| 33 | Shouldn’t the axes be labeled in some way? | In Figure 1, we have included labels for each of the axes. |
|  |  | We are grateful for your comments and suggestions that have helped to improve our manuscript. |
| **REVIEWER 3** | | |
| 34 | Less clear, however, are the ways in which the authors see this study’s findings advancing the field, and the extent to which the study is sufficiently distinct, as a secondary data analysis, from the primary parent study. | We have addressed these points in the following responses. |
| 35 | In the introduction, the authors describe two existing summary overviews of criticism of mixed-methods research but that, to date, no empirical examination of this domain has been carried out. While the fact that prior work, until now, has discussed this topic in terms of theory and methodology is notable, I am not sure that the authors have provided a convincing rationale yet for why this particular study is useful. This seems important to explicate, especially given the nearly complete overlap of the study’s findings with these two prior syntheses. | We have strengthened the rationale of our study on lines 89-107. In this paragraph, we have argued, first, that our study might “lead to valuable insights that could lay the basis for further discussion needed to ensure the healthy development of the field" and that “addressing those criticisms is also essential to enhance researchers' understanding of the complexity of the mixed methods field and to provide them with the necessary insights to deal with any tensions that might emerge when working in teams with researchers subscribing to different methodological viewpoints”. Second, we have pointed out that it would be useful to examine other researchers’ views on this topic because most of the criticisms relating to the mixed methods field have been formulated in the literature by a group of scholars dedicated to the theoretical aspects of methodology. Third, we have pointed out that, given the likely influence of social contexts on how researchers think about mixed methods, it would be appropriate to examine how the criticisms of the mixed methods field differ across disciplines.  Also, in the Discussion section, we have highlighted that: (1) we identified two new criticisms that had not been discussed previously in the literature (lines 632-664), and (2) some of the criticisms formulated by the participants were contradictory (lines 695-699). |
| 36 | Could the authors clarify how they inferred the methodological expertise of the participants? | We did not infer the methodological expertise of the participants. We directly asked them in the interview guide to describe their methodological knowledge and whether this was stronger in qualitative or quantitative research. This has been clarified in the data collection section (line 165 and Table 1). |
| 37 | From the authors’ stated inclusion criteria, participants from other disciplines that commonly employ mixed methods research (public health, social welfare, etc.) were excluded from the study. Though it may be unrealistic to expect a study like this to include participants from comprehensive disciplines, could the authors add some clarification on their defined focus on education, nursing, psychology, and sociology? | We have clarified and explained in detail our reasons for choosing these four disciplines (lines 139-146) |
| 38 | I recommend that the authors add clarification and explanation of how, exactly, the current study really diverges from the parent study such that it constitutes a qualitative secondary data analysis rather than a presentation of residual findings from the parent analysis already published. The authors may wish to reference Williams and Collins (2002) for an example of structuring an analysis section with more fidelity to the approach of qualitative secondary data analysis: Williams, C. C., & Collins, A. A. (2002). The social construction of disability in schizophrenia. Qualitative Health Research, 12(3), 297-309. At multiple points the authors highlight that the criticisms they are discussing are particularly relevant because they were given spontaneously in the original study. However, the original study appears to focus on evaluations of mixed methods research. This seems like a natural setting in which criticisms and approvals would be raised– all of which is to say, I am not sure that this is as noteworthy a characteristic of the current study as the authors seem to suggest it is. Indeed, at least two of the current study’s identified criticisms were already presented as findings in the original study. | While the original study was about the conceptualization and operationalization of the notion of quality in mixed methods research, this secondary analysis has its focus on the ways in which mixed methods have been developed and conceptualized by the mixed methods community. These are two different topics. We agree with the reviewer that our description of the purpose of our study was not sufficiently clear in the manuscript we submitted. Therefore, in several parts of the text, we have made it clear that the focus of our study is the researchers’ views of the development and conceptualization of the mixed methods approach by the mixed methods community (for instance, lines 188-191). Thank you also for suggesting the reference by Williams & Collins (2002). We have restructured the methods section using the study of these authors as a useful example. Now the distinction between the original study and the secondary analysis is made clear throughout the paper. |
| 39 | How transferable do the authors believe their findings are for other researchers who are mixed methodologists? (Keeping in mind that the study likely excluded researchers who work at the intersection of the inclusion criteria disciplines which may affect their views and experiences of mixed methods research.) | We have pointed out the limitations of the transferability of our findings by pointing out that while it is likely that researchers from other disciplines that also have a high prevalence of mixed methods studies have also formulated criticisms of their own, from our findings alone, it is not possible to infer how frequent these criticisms are and what types of criticisms may be more prevalent in each of the disciplines (lines 760-765). |
| 40 | I am not sure that it is clear yet how the current study provides much insight beyond what Creswell and others already have established. As the authors note in the discussion, the vast majority of the criticisms they identified have previously been documented in literature about mixed methods approaches to research. If the authors don’t identify much additional insight, I think that can be ok, as mounting evidence of the need for clarity among mixed-methods research could be seen as important for the continued growth, development, and advancement of the field. But if the authors see other novel conclusions, they should state this clearly. In the discussion section the authors dedicate the majority of space to reiterating that most of their findings have been noted in other research. There is comparatively less content discussing and exploring what their two new findings (lines 515-517) mean for the domain of mixed methods research. | We have included two new paragraphs to expand the explanation of the two new criticisms identified in our study (lines 632-664). |
| 41 | There seem to be some interesting contradictions present in the study’s findings that are not currently attended to or processed in the discussion section. For instance, the finding pertaining to needing consensus on terminology (e.g., lines 225-228) within criticism 2 seems at odds with criticism 11 in which it is described that “participants criticized a tendency in the mixed methods field to homogenize terminology.” Similarly, criticism 8 focuses on the notion that literature on mixed-methods presents typologies that “unnecessarily simplif[y] the process of carrying out a mixed methods study” and “curtail the creativity of researchers,” yet criticism 9 focuses on how researchers often do not align their mixed-methods practice with procedures documented in the literature. It may make sense that a diverse sample may have differing stances, but the discussion section would benefit from explicit attention to and interpretation of these incongruencies. | Thanks for raising this point. In the “Theoretical implications” section, we have pointed out these contradictions to exemplify the disagreements among mixed methods researchers on foundational issues, owing to differences in philosophical stances and their membership to different research cultures (lines 695-699). |
| 42 | Though the discussion section discusses some practical implications of the study’s findings, this section would benefit from additional detail and substance. For example, what do the authors recommend as requisite steps to building awareness (lines 588-591)? How, exactly, are the authors suggesting that procedural criticisms (lines 566-570) should be addressed in order to improve the practice of mixed methods research? | Since we have decided to restructure the discussion section to give more emphasis to theoretical aspects, we have removed this text fragment. |
| 43 | On page 7, “Questions from the background, (b) participants’ conceptualization of mixed methods research, and (c) how participants perceived the quality of the mixed methods approach in practice” is not a complete sentence. | Thanks for pointing this out. We added the fragment of text that was missing: “Questions from the interview guide were focused on the following: a) participant’s research background, (b) participant’s conceptualization of…” |
| 44 | In Table 2, 1.2., “proponent” should be “proponents”. | Thanks for pointing this out. We have corrected the word. |
| 45 | Criticisms as section headings should be phrased consistently either as the researchers’ belief or the belief they’re taking a stance against. For instance, criticism 4 is “Mixed methods research is not a third paradigm” and seems appropriately worded as a criticism; however, criticism 10 is “Mixed methods research is better than monomethod research” when the criticism itself seems to be that that mixed-methods research is not better than monomethod research. | Thanks for this comment. We have rephrased criticism 10 to reflect the researchers’ belief that mixed methods is not better than monomethod research. All criticisms are now consistently phrased as the researchers’ beliefs. |
|  |  | We are grateful for your comments and suggestions that have helped to improve our manuscript. |
